# Presentation and Outcomes of Kawasaki Disease in Latin American Infants Younger Than 6 Months of Age: A Multinational Multicenter Study of the REKAMLATINA Network

**DOI:** 10.3389/fped.2020.00384

**Published:** 2020-07-16

**Authors:** Elizabeth Moreno, S. Diana Garcia, Emelia Bainto, Andrea P. Salgado, Austin Parish, Benjamin D. Rosellini, Rolando Ulloa-Gutierrez, Luis M. Garrido-Garcia, Lourdes Dueñas, Dora Estripeaut, Kathia Luciani, Francisco J. Rodríguez-Quiroz, Olguita del Aguila, Germán Camacho-Moreno, Virgen Gómez, Tamara Viviani, Martha I. Alvarez-Olmos, Heloisa Helena de Souza Marques, Enrique Faugier-Fuentes, Patricia Saltigeral-Simental, Eduardo López-Medina, Greta Miño-León, Sandra Beltrán, Lucila Martínez-Medina, Maria C. Pirez, Fernanda Cofré, Adriana H. Tremoulet

**Affiliations:** ^1^California/Rady Children's Hospital San Diego, University of California, San Diego, San Diego, CA, United States; ^2^Pontificia Universidad Católica de Chile, Santiago, Chile; ^3^Meta-Research Innovation Center at Stanford University, Stanford, CA, United States; ^4^Servicio de Infectología, Hospital Nacional de Niños Dr. Carlos Sáenz Herrera, Centro de Ciencias Médicas, Caja Costarricense de Seguro Social (CCSS), San Jose, Costa Rica; ^5^Servicio de Cardiología, Instituto Nacional de Pediatría, Mexico City, Mexico; ^6^Servicio de Infectología, Hospital de Niños Benjamín Bloom, San Salvador, El Salvador; ^7^Servicio de Infectología, Hospital del Niño Dr. José Renán Esquivel, Panama City, Panama; ^8^Servicio de Infectología, Hospital de Especialidades Pediátricas Omar Torrijos Herrera, Caja de Seguro Social, Panama City, Panama; ^9^Servicio de Reumatología, Instituto Hondureño de Seguridad Social, Tegucigalpa, Honduras; ^10^Unidad de Infectología Pediátrica, Hospital Nacional Edgardo Rebagliati Martins, Lima, Peru; ^11^Servicio de Infectología, Fundación HOMI Hospital Pediátrico de la Misericordia and Universidad Nacional de Colombia, Bogota, Colombia; ^12^Servicio de Infectología, Centro Médico Universidad Central del Este, Santo Domingo, Dominican Republic; ^13^Servicio de Infectología, Hospital Sotero del Río, Santiago, Chile; ^14^Servicio de Infectología, Fundación Cardioinfantil and Universidad El Bosque, Bogota, Colombia; ^15^Servicio de Infectología, Hospital Das Clinicas da Faculdade Medicina da USP, São Paulo, Brazil; ^16^Servicio de Reumatología, Hospital Infantil de México Federico Gómez, Mexico City, Mexico; ^17^Servicio de Infectología, Hospital Infantil Privado & Instituto Nacional de Pediatría, Mexico City, Mexico; ^18^Servicio de Infectología, Hospital Universitario del Valle & Centro Médico Imbanaco and Clínica Farallones, Cali, Colombia; ^19^Servicio de Infectología, Hospital del Niño “Dr. Francisco de Icaza Bustamante”, Guayaquil, Ecuador; ^20^Servicio de Infectología, Clínica Colsanitas, Bogota, Colombia; ^21^Servicio de Infectología, Centenario Hospital Miguel Hidalgo, Aguascalientes, Mexico; ^22^Servicio de Infectología, Hospital Pediátrico Centro Hospitalario Pereira Rossell, Montevideo, Uruguay; ^23^Servicio de Infectología, Hospital Roberto del Río, Santiago, Chile

**Keywords:** Kawasaki disease, Latin America, infants, coronary artery abnormalities, delayed diagnosis

## Abstract

**Objective:** To characterize the clinical presentation and outcomes of Kawasaki disease (KD) in infants <6 months of age as compared to those ≥6 months in Latin America.

**Methods:** We evaluated 36 infants <6 months old and 940 infants ≥6 months old diagnosed with KD in Latin America. We compared differences in laboratory data, clinical presentation, treatment response, and coronary artery outcomes between the two cohorts.

**Results:** The majority (78.1%) of infants and children ≥6 months of age were initially diagnosed with KD, as compared to only 38.2% of infants <6 months. Clinical features of KD were more commonly observed in the older cohort: oral changes (92 vs. 75%, *P* = 0.0023), extremity changes (74.6 vs. 57.1%, *P* = 0.029), and cervical lymphadenopathy (67.6 vs. 37.1%, *P* = 0.0004). Whether treated in the first 10 days of illness or after the 10th day, infants <6 months were at greater risk of developing a coronary artery aneurysm compared to KD patients ≥6 months treated at the same point in the course of illness [ ≤ 10 days (53.8 vs. 9.4%, *P* = 0.00012); >10 days (50 vs. 7.4%, *P* = 0.043)].

**Conclusion:** Our data show that despite treatment in the first 10 days of illness, infants <6 months of age in Latin America have a higher risk of developing a coronary artery aneurysm. Delay in the diagnosis leads to larger coronary artery aneurysms disproportionately in these infants. Thus, suspicion for KD should be high in this vulnerable population.

## Introduction

Kawasaki disease (KD) is a medium-size vessel vasculitis of childhood with a clinical presentation that can be confused with other pediatric febrile illnesses. This can lead to a delay in diagnosis and treatment with intravenous immunoglobulin (IVIG), which in turn can lead to a higher rate of coronary artery abnormalities (CAA) and increased mortality and morbidity (1–6). Despite timely treatment with IVIG within the first 10 days of illness, up to 43.4% of infants <6 months in the United States have been reported to develop CAA (3).

Most of the epidemiological data for patients with KD <6 month of age are from regions outside of Latin America (LA) (2, 6–11). It is unknown whether the clinical presentation and outcomes are similar in infants <6 months in LA populations. Increasing collaborative academic and clinical efforts in Latin America to study the epidemiology and care of KD patients led to the development of a multinational network in LA entitled Red de Enfermedad de Kawasaki en America Latina (REKAMLATINA; Latin American KD Network) (8, 12). We aim to characterize the clinical presentation and outcomes of KD in infants <6 months old as compared to those ≥6 months of age in LA.

## Materials and Methods

### Subjects and Clinical Data

All study data were obtained from review of the REKAMLATINA Research Electronic Data Capture (REDCap) database, housed at the University of California San Diego KD Research Center, which contains demographic, clinical and laboratory data from KD patients throughout LA. We reviewed retrospectively collected data from 36 KD subjects <6 months and 940 ≥6 months diagnosed and treated in 16 Latin American countries and 35 hospitals between January 1, 2009, to December 31, 2013.

In accordance with the American Heart Association (AHA) guidelines, complete KD presentation was defined as fever ≥ 5 days and 4 or more of the following clinical signs: erythema and cracking of lips, strawberry tongue, and/or erythema of oral and pharyngeal mucosa; bilateral bulbar conjunctival injection without exudate; polymorphous skin rash; changes such as edema, redness, and/or peeling of the hands or feet; and cervical lymphadenopathy (13). Incomplete KD was defined per the AHA guidelines as fever and fewer than 4 of the KD clinical criteria with either supportive laboratory or an echocardiographic abnormality. Illness day 1 is defined as the first day of fever. Specifics on the treatment course, including treatment with IVIG or antibiotics, was collected as was the presence of IVIG resistance defined as persistent fever (T ≥ 38.0°C rectally or orally) > 36 h but less than 7 days after completion of the first IVIG infusion.

CAAs were classified as normal (Z < 2.5), dilated (Z ≥ 2.5 to ≤ 4), aneurysmal (Z > 4 to ≤ 10), or giant aneurysm (Z > 10) as per the 2004 AHA KD Guidelines by the local site study investigator, given that these were the published guidelines at the time of acquisition of the data (3, 14). For a subset in whom CAA measurements, height and weight were available (*N* = 481), the internal diameter normalized for body surface area (Z-score) of the proximal right coronary artery (RCA) and left anterior descending artery (LAD) were calculated. Height and weight were verified using age-appropriate normal values for males and females and the body surface area was calculated using the Haycock formula. Z-score was calculated using the formula from Dallaire and Dahdah (15). The maximal Z-score of either the RCA or LAD (Z-max) was identified as the largest Z-score for the subjects within the first 6 weeks of illness. The study received Institutional Review Board approval at the University of California, San Diego as well as at each individual institution enrolling subjects in the REKAMLATINA database.

### Statistical Methods

The primary comparisons were made between infants <6 months old and infants and children ≥6 months old. Differences in counts and categorical variables were compared using two-sided Fisher's exact test. Continuous variables were compared using the Mann-Whitney test. Statistical processing was performed using R version 3.1.0 (available at: http://www.R-project.org) and GraphPad Prism version 8.2.1 (available at: (https://www.graphpad.com).

## Results

Over a five-year period, we identified a total of 976 patients <13 years of age with KD in LA. Retrospectively collected demographic and clinical data are presented in Table 1. Of the 976 patients, 36 (3.7%) were <6 months of age. The remaining 940 patients (96.3%) were between 6 months and 12 years of age (Table 1). Most of the children <6 months (25 of 36, 69.4%) were described as white (*P* = 0.0059). Most subjects were male and there was no difference in sex by age cohort (72.2 vs. 60.9%, *P* = 0.22). As compared to subjects ≥6 months old, infants <6 months had an illness day at hospitalization of 4 days as compared to illness day 6 (*P* = 0.00018). The younger cohort had longer hospital stays on average (8 vs. 5 days, *P* < 0.0001).

**Table 1:** Demographic and clinical characteristics of the study population^a^.

**Characteristic**	**<6 months old^b^ (*N* = 36)**	**≥6 months old^b^ (*N* = 940)**	***p*-value**
Age (months)	4.0 (3.0 to 5.0)	26.0 (16 to 48.0)	<0.0001^*^
Illness day at hospitalization (days)	4.0 (3.0 to 7.0)	6.0 (5.0 to 9.0)	0.00018^*^
Hospital stay (days)	8.0 (6.0 to 10.0)	5.0 (4.0 to 7.0)	<0.0001^*^
Male sex, *N* (%)	26 (72.2%)	572 (60.9%)	0.22
Race/Ethnicity, *N* (%)			
White	25 (69.4%)	427 (45.5%)	0.0059^*^
Asian	1 (2.8%)	4 (0.4%)	0.17
Afro-Latino	2 (5.6%)	28 (3.0%)	0.31
Indigenous	1 (2.8%)	40 (4.3%)	>0.99
Unknown	7 (19.4%)	439 (46.8%)	0.0010^*^
WBC (x10^3^)	18.6 (15.7 to 23.2)	14.1 (10.9 to 18.0)	<0.0001^*^
Neutrophils	9.4 (7.0 to 12.5)	8.8 (5.9 to 12.1)	0.52
Hemoglobin z-score^c^	−1.4 (-2.2 to−0.29)	−1.2 (-2.3 to 0.17)	0.63
Platelets (x10^3^)	439 (311 to 549)	400 (294 to 521)	0.52
ESR (mm/h)	56 (36 to 81)	46 (30 to 56)	0.21
CRP (mg/dl)	6.0 (1.9 to 9.7)	6.4 (2.4 to 12.1)	0.51
ALT (U/L)	22.0 (16.7 to 30.0)	40.0 (22.0 to 85.0)	0.00028^*^
GGT (U/L)	24.0 (18 to 61.0)	45.0 (22.5 to 120.8)	0.35
Albumin	3.1 (3.0 to 3.6)	3.3 (2.9 to 3.7)	0.94

a *All continuous values were expressed as median, with 25 to 75% IQR. Differences in continuous values were tested using Mann-Whitney test. Differences in count values were assessed using Fisher's exact test, two-sided*.

b *We had complete data except for each of the following variables: Illness day N = 961 (<6mo = 35, ≥6mo = 926), Hospital stay N = 955 (<6mo = 35, ≥6mo = 920), Race/Ethnicity N = 974 (<6mo = 36, ≥6mo = 938), WBC N = 939 (<6mo = 31, ≥6mo = 908), Neutrophils N = 894 (<6mo = 27, ≥6mo = 867), Hb Z score N = 947 (<6mo = 31, ≥6mo = 916), Platelets N = 938 (<6mo = 32, ≥6mo = 906), ESR N = 619 (<6mo = 10, ≥6mo = 609), CRP N = 829 (<6mo = 31, ≥6mo = 798), Albumin N = 597(<6mo = 22, ≥6mo = 575), ALT N = 791(<6mo = 21, ≥6mo = 770), GGT N = 157(<6mo = 5, ≥6mo = 152)*.

c *Hemoglobin z-score is distance in SD units from the mean for age-adjusted hemoglobin values*.

* *Indicates statistical significance (p < 0.05)*.

Infants <6 months had a higher mean white blood cell count at diagnosis (18.6 vs. 14.1, *P* < 0.0001; Table 1). Other inflammatory markers such as neutrophils, ESR, and platelets were not significantly different between the two age groups. The younger cohort had lower levels of ALT, GGT, and albumin, but only the ALT difference was statistically significant between the age groups (22 vs. 40 U/L, *P* = 0.00028).

Infants <6 months were less likely to be initially diagnosed with KD (38.2 vs. 78.1%, OR = 0.17, 95% CI 0.084 to 0.35, *P* < 0.0001) and had an 11-fold increased risk of being diagnosed initially with sepsis or shock (26.5 vs. 2.4%, OR = 14.5, 95% CI 6.0 to 33.8, *P* < 0.0001) (Table 2). Furthermore, infants <6 months were 4 times more likely to be initially diagnosed with a urinary tract infection (14.7 vs. 3.8%, OR = 4.4, 95% CI 1.7 to 11.5, *P* = 0.011) and more likely to be suspected of occult bacteremia initially (11.4 vs. 2.3%, OR = 5.4, 95% CI 1.9 to 15.7, *P* = 0.012) as compared to older infants and children.

**Table 2:** Initial clinical diagnosis on admission.

**Diagnosis^a^**	**<6 months old *N* (%)**	**≥6 months old *N* (%)**	***p*-value and OR (95% CI)**
Kawasaki disease	13 (38.2%)	711 (78.1%)	<0.0001^*^OR = 0.17 (0.084 to 0.35)
Scarlet fever	1 (2.9%)	36 (4.4%)	>0.99
Scalded skin syndrome	0 (0.0%)	9 (1.1%)	-
Sepsis/shock	9 (26.5%)	20 (2.4%)	<0.0001^*^ OR = 14.5 (6.0 to 33.8)
Erythema multiforme/SJS	1 (2.9%)	13 (1.6%)	0.45
Urinary tract infection	5 (14.7%)	31 (3.8%)	0.011^*^ OR = 4.4 (1.7 to 11.5)
Dengue	1 (2.9%)	20 (2.4%)	0.58
Adenovirus	0 (0.0%)	7 (0.9%)	-
Enterovirus	0 (0.0%)	6 (0.7%)	-
Other virus	2 (5.9%)	38 (4.6%)	0.67
Unspecified fever	9 (26.5%)	188 (22.6%)	0.68
Occult bacteremia	4 (11.4%)	19 (2.3%)	0.012^*^ OR = 5.4 (1.9 to 15.7)

a *Some subjects were classified with more than one diagnosis or, in some other cases, a clinical diagnosis was not recorded on admission*.

* *Indicates statistical significance (p < 0.05)*.

Table 3 describes clinical findings related to diagnostic criteria for KD. Oral changes, including erythematous oropharynx or lips or strawberry tongue, were less commonly noted in the younger patients (75 vs. 92%, OR = 0.26, 95% CI 0.12 to 0.58, *P* = 0.0023). Cervical lymphadenopathy was also noted less frequently in younger patients (37.1 vs. 67.6%, OR = 0.28, 95% CI 0.14 to 0.55, *P* = 0.0004), as were extremity changes (57.1 vs. 74.6%, OR = 0.45, 95% CI 0.23 to 0.88, *P* = 0.029). Subjects were classified as having complete vs incomplete KD, based on AHA 2017 guidelines (13). Overall, we found significantly more older infants and children presenting with complete KD (44.4 vs. 76.6%, OR = 0.24, 95% CI 0.13 to 0.47, *P* < 0.0001). The younger cohort had over 4-fold greater odds of being diagnosed as incomplete KD compared to their older counterparts (55.6 vs. 23.4%, OR = 4.1, 95% CI 2.1 to 8.0, *P* < 0.0001).

**Table 3:** Diagnostic criteria for KD.

**Clinical Presentation**	**<6 months old *N* (%)**	**≥6 months old *N* (%)**	***p*-value^d^ and OR (95% CI)**
Rash	30 (83.3%)	814 (87.2%)	0.45
Conjunctival injection	29 (80.6%)	812 (86.9%)	0.31
Oral changes^a^	27 (75%)	865 (92%)	0.0023^*^ OR = 0.26 (0.12 to 0.58)
Cervical lymphadenopathy^a^	13 (37.1%)	627 (67.6%)	0.0004^*^ OR = 0.28 (0.14 to 0.55)
Extremity changes	20 (57.1%)	694 (74.6%)	0.029^*^ OR = 0.45 (0.23 to 0.88)
Complete KD^b^, *N* (%)	16 (44.4%)	720 (76.6%)	<0.0001^*^ OR = 0.24 (0.13 to 0.47)
Incomplete KD^c^, *N* (%)	20 (55.6%)	220 (23.4%)	<0.0001^*^ OR = 4.1 (2.1 to 8.0)

a *These clinical features are based on the American Heart Association criteria for KD. Oral changes include erythematous oropharynx or lips or strawberry tongue. Cervical lymphadenopathy is unilateral and is a lymph node at least 1.5 cm*.

b *Complete KD as defined by the AHA with at least 4 of the 5 clinical criteria*.

c *Incomplete KD by laboratory evaluation is defined the AHA with <4 clinical criteria and laboratory inflammation (ESR ≥ 40 mm/hr or CRP ≥ 3 mg/dl with ≥ 3 supplementary labs elevated: albumin levels of <3.0 g/dL, anemia for age, elevation of ALT level, >450,000 platelets per mm^3^ after the seventh day, white blood cell count of >15,000 cells per mm^3^, and >10 white blood cells per high-power field in the urine)*.

d *Fisher's exact test was used to test differences in count data*.

* *Indicates statistical significance (p < 0.05)*.

Table 4 describes IVIG therapy as well as use of antibiotics. The initial use of antibiotics and the treatment of patients ultimately diagnosed with KD with IVIG was high in both groups, though not statistically different between the cohorts.

**Table 4:** IVIG therapy course, IVIG-resistance and rate of antibiotic use^*^.

	**<6 months old**	**≥6 months old**
Treated with IVIG, N (%)^a^	35/36 (97.2%)	879/937 (93.8%)
Treated with Antibiotics prior to KD diagnosis	26/35 (74.3%)	626/907 (69.0%)

a *All subjects who received IVIG were given a 2 g/kg dose*.

b *Fisher's exact test*.

* *All p-values are >0.05 and thus not significant*.

Table 5 describes differences in the Z-max of the left anterior descending and right coronary arteries in young vs older KD patients by illness day. For children hospitalized in the first 10 days of illness, infants <6 months increased the aneurysm risk 6-fold compared to those subjects ≥6 months old (53.8 vs. 9.4%, OR = 11.25, 95% Cl 3.87 to 36.25, *P* = 0.00012). Infants <6 months hospitalized after day 10 of illness had a greater risk of having a giant coronary artery aneurysm as compared to older children (50 vs. 7.4%, OR = 12.50, 95% CI 1.62 to 84.97, *P* = 0.043). Infants <6 months had a higher baseline Z-score and Z-max compared to older patients, regardless of whether they were hospitalized in the first 10 days or after the 10th day of illness. When diagnosed within the first 10 days of illness, younger patients had a higher baseline Z-score and Z-max compared to older patients (2.37 vs. 0.77, *P* = 0.0059; 4.55 vs. 1.18, *P* = 0.0022), respectively. An even greater baseline Z-score and Z-max was observed for younger infants after the 10th day of illness (10.35 vs. 1.65, *P* = 0.0064; 8.04 vs. 1.86, *P* = 0.0045).

**Table 5:** Coronary artery abnormalities in KD subjects treated (All)^a^^,^^d^^,^^e^.

**Coronary artery**	**<6 months old *N* (%)**	**≥6 months old *N* (%)**	***p*-value**	**OR (95% CI)**
**CLASSIFICATION BASED ON Z-MAX SCORE, EITHER LAD OR RCA**				
**Illness Day** **<** **=** **10 days**				
Normal^b^	5/13 (38.5%)	307/383 (80.2%)	0.0015^*^	0.15 (0.056 to 0.51)
Dilated Z-score ≥2.5 to ≤4	1/13 (7.7%)	33/383 (8.6%)	>0.9999	0.88 (0.080 to 5.14)
Aneurysmal Z > 4 to ≤ 10	7/13 (53.8%)	36/383 (9.4%)	0.00012^*^	11.25 (3.87 to 36.25)
Giant Aneurysm Z>10	0/13 (0.0%)	7/383 (1.8%)	>0.9999	-
First echo Z-score	2.37 (1.02 to 5.08)	0.77 (−0.03 to 1.72)	0.0059^*^	-
Z-max^c^	4.55 (1.09 to 5.95)	1.18 (0.19 to 2.07)	0.0022^*^	-
**Illness Day** **>** **10 days**				
Normal^b^	0/4 (0.0%)	49/81 (60.5%)	0.03^*^	-
Dilated Z-score ≥2.5 to ≤4	0/4 (0.0%)	9/81 (11.1%)	>0.9999	-
Aneurysmal Z > 4 to ≤ 10	2/4 (50.0%)	17/81 (21.0%)	0.21	3.77 (0.55 to 24.87)
Giant Aneurysm Z>10	2/4 (50.0%)	6/81 (7.4%)	0.043^*^	12.50 (1.62 to 84.97)
First Echo Z-score	10.35 (5.73 to 14.50)	1.65 (0.31 to 4.31)	0.0064^*^	-
Z-max^c^	8.04 (5.32 to 15.38)	1.86 (0.54 to 4.38)	0.0045^*^	-

a *Data expressed as N (%) or median (IQR); Fisher's exact test, two sided*.

b *Subjects were classified as having normal (<2.5 standard deviation units [Z-score] from the mean, normalized for body surface area), dilated (Z-score ≥2.5 to ≤4), or aneurysmal (Z > 4 to ≤ 10 for giant aneurysm) coronary arteries on the basis of the maximal internal diameters of the right coronary artery (RCA) and left anterior descending artery (LAD) measured by echocardiography at the time of diagnosis and up to 8 weeks after onset of fever. Coronary artery abnormalities were classified as normal, dilated or aneurysmal (focal dilation of an arterial segment at least 1.5 times the diameter of the adjacent segment) based on the AHA 2004 KD Guidelines, as those were the published guidelines at the time of data collection*.

c *Z max defined as largest Z score of LAD or RCA in the first 6 weeks of illness*.

d *Unavailable CAA data for N = 495 (<6mo = 19, ≥6mo = 476)*.

e *Available Z-score data on N = 489 (<6mo = 18, ≥6mo = 471), however, only Z-scores data on N = 481 (<6mo = 17, ≥6mo = 464) were used for CAA analysis. Subjects with Z-scores, but without a verified height and weight or illness day were excluded from the CAA analysis*.

* *Indicates statistical significance (p < 0.05)*.

## Discussion

This is the first study to compare the clinical presentation, initial diagnosis and treatment, and coronary artery outcomes between infants <6 months compared to those 6 months and older throughout LA. As reflected in other recent studies, infants <6 months old with acute KD in LA are more likely to develop CAA than those older than 6 months old (2, 3, 6, 9, 16–18). In this study, based on calculated Z-max scores, 53.8% of infants <6 months with KD had an aneurysm or giant aneurysm compared to the older cohort 11.2%. By comparison, other studies have found ~20% of infants <6 months developed an aneurysm or great aneurysm, whereas only 5% of infants ≥6 months had an aneurysm or great aneurysm (3). In the Rosenfeld et al. study the CAA present in infants <6 months was as high as 79%, compared with 44% of infants ≥6 months (6). Similarly to LA, a high prevalence of coronary artery abnormalities in infants <6 months has been observed in other parts of the globe. In a study from Chandigarh, India it was reported 35% of infants <6 months had coronary artery abnormalities and 65% in a study in Taipei, Taiwan (2, 18). While the increased rate in this cohort may be due to Z-scores only being available in the most severely affected patients, it does warrant assessing whether there is in fact a higher rate of aneurysms in infants in LA in a follow up cohort study. Furthermore, as per the recommendations of the 2017 AHA guidelines, these higher risk infants may benefit from adjunctive therapy.

A novel data-point captured in our database was “Initial Diagnosis.” KD was the initial diagnosis in 74.2% of all subjects, but only in 38.2% of the infants <6 months, as sepsis and a urinary tract infection were the most common initial diagnoses. With nearly all patients being initially treated with antibiotics, it is important to continue to raise awareness that KD is not a diagnosis of exclusion and that in many cases antibiotics are not needed as KD alone is the leading diagnosis.

There are several strengths and limitations to our study. These data are from the largest international network and database of children with KD and provide the demographic and clinical data from an area of the world where little has been published widely about KD. That said, there were a larger proportion of infants and children ≥6 months as compared to those <6 months. For this specific retrospective database, illness day at diagnosis of KD was not available as some sites considered this to be protected health information. Instead illness day at hospitalization was recorded, keeping in mind that in some cases KD was not the initial diagnosis. In addition, Z-score could only be calculated in subjects in whom an accurate weight and height were available for calculating BSA.

## Conclusions

In summary, our study shows that despite treatment in the first 10 days of illness, infants <6 months old in LA have a higher risk of developing a coronary artery aneurysm than older KD patients. Delay in the diagnosis leads to larger coronary artery aneurysms disproportionately in these infants. In addition, the majority of infants ultimately diagnosed with KD are initially thought to have an infectious issue initially and thus treated with antibiotics. Thus, the suspicion for KD should be high in infants <6 months old given the likelihood of misdiagnosis and increased risk for coronary artery aneurysm formation.

## Data Availability Statement

The datasets generated for this study are available on request to the corresponding author.

## Ethics Statement

The studies involving human participants were reviewed and approved by Institutional Review Board at the University of California, San Diego and by the ethics committee at each REKAMLATINA-2 Study participant site. Written informed consent from the participants' legal guardian/next of kin was not required to participate in this study in accordance with the national legislation and the institutional requirements.

## Author's Note

Presented in part as an abstract at the *9th World Congress of the World Society for Pediatric Infectious Diseases (WSPID 2015)*. Rio de Janeiro, Brasil. November 18-21, 2015 (19).

## Author Contributions

AS, RU-G, and AT developed the concept for this project. EM, SG, EB, AP, and AT were responsible for data cleaning and analysis. AT provided mentorship to SG, EM, and AP throughout the study period. All authors contributed to the article and approved the submitted version.

## The REKAMLATINA-2 Study Group Investigators

Lorena Franco, Nora Bueno (Hospital Infantil Municipal de Córdoba, Córdoba, Argentina), Jaime Deseda-Tous (Hospital Español Auxilio Mutuo, San Juan, Puerto Rico), Carlos F. Grazioso, Pablo J. Grazioso (Sanatorio Nuestra Sra. Del Pilar/Hospital General San Juan de Dios, Ciudad Guatemala, Guatemala), Mariella Vargas-Gutierrez, Susan Li-Chan, Maria L. Avila-Agüero, Kattia Camacho-Badilla, Alejandra Soriano-Fallas (Hospital Nacional de Niños “Dr. Carlos Sáenz Herrera, Centro de Ciencias Médicas de la Caja Costarricense de Seguro Social, San José, Costa Rica), Paola Pérez-Camacho (Fundación Valle del Lili, Cali, Colombia); Luisa B. Gámez-González (Hospital Infantil de Chihuahua, Chihuahua, México), Giannina Izquierdo, Pilar Picart (Hospital de Niños “Dr. Exequiel González Cortés, Santiago, Chile), Adrián Collia, Alejandro Ellis (Sanatorio Mater Dei, Buenos Aires, Argentina), Maria del Carmen Luis-Álvarez (Hospital Pediátrico Universitario “William Soler,” La Habana, Cuba); Stella Gutierrez, Estefanía Fynn, Elizabeth Assandri (Hospital CASMU, Montevideo, Uruguay), Mario Melgar (Hospital Roosevelt, Ciudad Guatemala, Guatemala), Carlos Daza (Hospital Materno Infantil José Domingo de Obaldía), Jacqueline Levy (Hospital del Niño Dr. José Renán Esquivel, Ciudad Panamá, Panamá), Isabel C. Hurtado-Palacios (Hospital Universitario del Valle, Centro Médico Imbanaco, Cali, Colombia), Angélica Calvache-Burbano, Antonio Fernández, Nelly Chávez-Solórzano, Marianella Layana-Coronel, Denisse Olaya-González (Hospital del Niño “Dr. Francisco de Icaza Bustamante, Guayaquil, Ecuador), Marco A. Yamazaki-Nakashimada, Raymundo Rodríguez-Herrera (Instituto Nacional de Pediatría, Ciudad de México, México), Sarbelio Moreno-Espinosa, Ángel Flores (Hospital Infantil de México Federico Gómez, Ciudad de México, México), Adriana Díaz-Maldonado, Kelly Marquez-Herrera, Roy Sanguino-Lobo (Fundación HOMI Hospital Pediátrico de la Misericordia; Bogotá, Colombia), Natalia Lara (Universidad Nacional de Colombia & Hospital de la Misericordia, Bogotá, Colombia), Diana López-Gallegos (Hospital Infantil Privado, Ciudad de México, México), Neusa Keico Sakita, María Fernanda Pereira Badue, Gabriela Leal (Hospital Das Clinicas da Faculdade Medicina da USP, Sao Paolo, Brazil), Diana C. Medina, Paula Araque (Fundación Cardioinfantil & Universidad El Bosque, Bogotá, Colombia), Pilar Guarnizo, Claudia Stapper, Manuel Huertas-Quiñones, María Fernanda García-Venegas (Fundación Cardioinfantil, Universidad Nacional de Colombia y Universidad del Rosario, Bogotá, Colombia), Pio López (Hospital Universitario del Valle, Cali, Colombia), Mónica Pujadas, Karina Machado, Federica Badía, Alejandra Vomero (Hospital Pediátrico Centro Hospitalario Pereira Rossell, Montevideo, Uruguay), Jaime Patiño, Daniela Cleves (Fundación Valle del Lili, Cali, Colombia), Margarita Martínez-Cruzado (Hospital Español Auxilio Mutuo, San Juan, Puerto Rico), Mario Gamero (Hospital de Niños Benjamín Bloom; San Salvador, El Salvador), Guillermo Soza, Carolina Cerda (Hospital Dr. Hernán Enríquez Aravena, Temuco, Chile), Sergio Bernal-Granillo (Hospital General de Zona 1/IMMS/Hospital Ángeles CMP, San Luis Potosí, México), Belén Amorín (Hospital Escuela del Litoral Paysandú, Paysandú, Uruguay).

## Conflict of Interest

The authors declare that the research was conducted in the absence of any commercial or financial relationships that could be construed as a potential conflict of interest.
